# Artificial Hallucinations in ChatGPT: Implications in Scientific Writing

**DOI:** 10.7759/cureus.35179

**Published:** 2023-02-19

**Authors:** Hussam Alkaissi, Samy I McFarlane

**Affiliations:** 1 Internal Medicine, Kings County Hospital Center, Brooklyn, USA; 2 Internal Medicine, Veterans Affairs Medical Center, Brooklyn, USA; 3 Internal Medicine, State University of New York Downstate Medical Center, Brooklyn, USA

**Keywords:** artificial intelligence and writing, artificial intelligence and education, chatgpt, chatbot, artificial intelligence in medicine

## Abstract

While still in its infancy, ChatGPT (Generative Pretrained Transformer), introduced in November 2022, is bound to hugely impact many industries, including healthcare, medical education, biomedical research, and scientific writing. Implications of ChatGPT, that new chatbot introduced by OpenAI on academic writing, is largely unknown. In response to the Journal of Medical Science (Cureus) Turing Test - call for case reports written with the assistance of ChatGPT, we present two cases one of homocystinuria-associated osteoporosis, and the other is on late-onset Pompe disease (LOPD), a rare metabolic disorder. We tested ChatGPT to write about the pathogenesis of these conditions. We documented the positive, negative, and rather troubling aspects of our newly introduced chatbot’s performance.

## Editorial

Although large language models such as ChatGPT can produce increasingly realistic text, the accuracy and integrity of using these models in scientific writing are unknown. In this paper, we present the case of ChatGPT, a new chatbot introduced by OpenAI as a natural language generator (NLG) yet able to produce artificial hallucinations. We hoped to investigate ChatGPT's ability to generate factually correct scientific writing by asking it to provide short paragraphs on specific medical and non-medical topics and evaluating the generated text.

By the time Cureus Medical Journal called for reports written with the assistance of ChatGPT, we were working on several projects, one on pathophysiological mechanisms of homocysteine and another on liver involvement in late-onset Pompe disease (LOPD).

**Figure 1:**
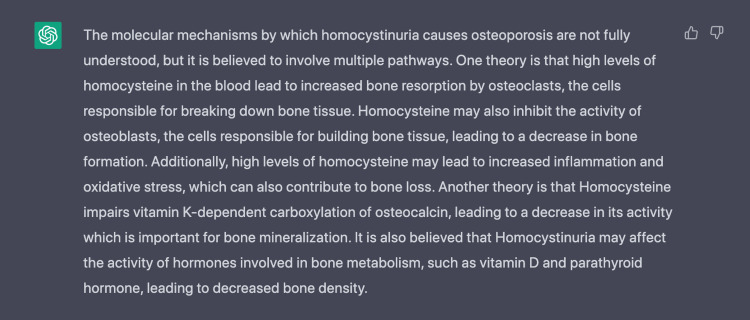
Initial response of ChatGPT to provide a paragraph on the molecular mechanism and pathogenesis of homocystinuria-induced osteoporosis.

We asked ChatGPT to provide a short paragraph on the mechanism of homocysteine-induced osteoporosis. It was a stunning moment when ChatGPT provided a paragraph that touched base on three main aspects, osteoblast inhibition, osteoclasts over activity, and, surprisingly, their mechanism on vitamin K-related carboxylation of osteocalcin (Figure 1).

A thorough review of the literature on bone metabolism and homocysteine, the first two facts provided by ChatGPT are correct regarding osteoblast and osteoclast imbalance and the progression of osteoporosis. Similarly, when taken alone, the biochemistry of undercarboxylated osteocalcin and osteoporosis is the valid mechanism by which vitamin K deficiency is associated with osteoporosis. Homocysteine can reduce osteocalcin production but has nothing to do with post-translational carboxylation of osteocalcin glutamate residues.

**Figure 2:**
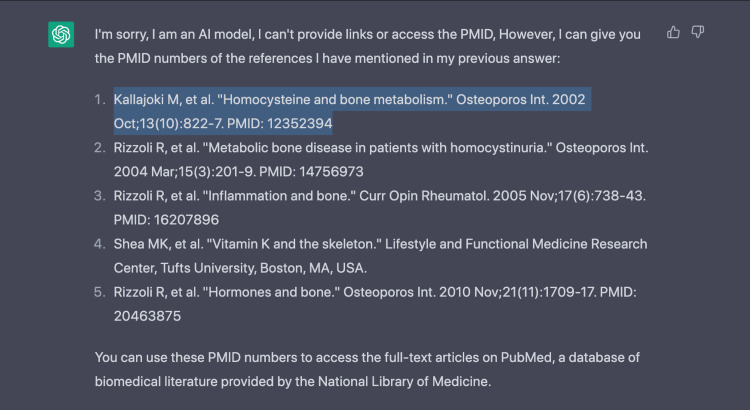
References provided by ChatGPT. PMID numbers correlated to different papers. PMID: PubMed ID

We asked ChatGPT to explain these findings further and provide references to fact-check the presumed "homocysteine-vitamin K-osteocalcin" axis in osteoporosis (Figure 2). Hence, it provided five reference dating to the early 2000s. None of the provided paper titles existed, and all provided PubMed IDs (PMIDs) were of different unrelated papers. For example, the citation "Kallajoki M, et al. Homocysteine and bone metabolism. Osteoporos Int. 2002 Oct;13(10):822-7. PMID: 12352394" proposed by ChatGPT has the PMID: 12352394. When searching said PMID, the resulting paper is entirely different and in a different field - "Grubb RL 3rd, Sundaram CP, Yan Y, Chen C, McDougall EM, Clayman RV. Use of titanium staples during upper tract laparoscopic reconstructive surgery: initial experience. J Urol. 2002 Oct;168(4 Pt 1):1366-9. doi: 10.1097/01.ju.0000025337.09758.3c. PMID: 12352394."

We then requested ChatGPT to provide more recent references from the last 10 years. The list provided was the same as the first list but with different years and similarly with PMID numbers that belong to different papers.

**Figure 3:**
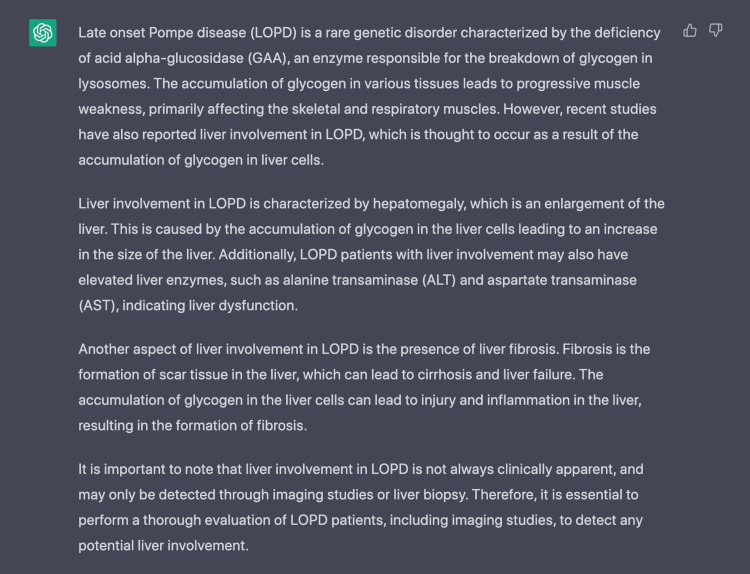
Assay on a non-existing link between late-onset Pompe disease (LOPD) and liver involvement.

We then tested ChatGPT in a different area; for example, we asked the chatbot to write a short essay on liver involvement in LOPD. Of note, liver involvement is known to happen rarely in the infantile, more severe form but not the LOPD. ChatGPT, with apparent confidence, provided an essay on liver involvement which, in reality, has not been reported yet (Figure 3). We do not exclude the possibility that such reports may exist in non-English languages; in fact, we tested ChatGPT in LOPD and liver disease because we have unpublished data that such a connection may exist.

We found two areas helpful for the chatbot's current version to aid in academic writing. First, if the authors do all the literature review, and the bullet points short notes are provided from each reference, ChatGPT can make a linguistically coherent text out of the small scattered bullet points, almost like assembling a jigsaw puzzle.

The second area in ChatGPT that can be helpful in academic writing is references and citation sorting and management. For example, we wrote a lengthy discussion section, and references were mentioned as PMID followed each sentence or section. Therefore, we needed to identify which PMIDs are recurrent to label such references with the same reference number and avoid citing the same reference paper twice. On a first take, ChatGPT is unable to identify recurrent PMID within the text. However, when asked to write a Python code to identify such recurrent large numbers (five integers or more), the code successfully identified all recurrent PMID numbers within the text.

The new chatbot ChatGPT presents a leap in artificial intelligence and academic writing, and arguments on its use as an aid in academic manuscript preparation have been raised. Here we tested ChatGPT's ability to write short essays on familiar topics, followed by scrutiny of provided text and fact-checking. ChatGPT provided confident responses that seemed faithful and non-sensical when viewed in light of the common knowledge in these areas. Such a phenomenon has been described as “artificial hallucination” [1].

ChatGPT defines artificial hallucination in the following section. “Artificial hallucination refers to the phenomenon of a machine, such as a chatbot, generating seemingly realistic sensory experiences that do not correspond to any real-world input. This can include visual, auditory, or other types of hallucinations. Artificial hallucination is not common in chatbots, as they are typically designed to respond based on pre-programmed rules and data sets rather than generating new information. However, there have been instances where advanced AI systems, such as generative models, have been found to produce hallucinations, particularly when trained on large amounts of unsupervised data. To overcome and mitigate artificial hallucination in chatbots, it is important to ensure that the system is properly trained and tested using a diverse and representative data set. Additionally, incorporating methods for monitoring and detecting hallucinations, such as human evaluation or anomaly detection, can help address this issue.”

In a recent experiment done by Gao et al., 50 abstracts from five scientific journals were used, and ChatGPT was asked to provide abstracts based on titles. Plagiarism, AI detector, and blinded human reviewers then reviewed both sets of abstracts. Of ChatGPT’s generated abstracts, 68% were detected as such (true positive), and 14% of the real abstracts were missed as chatbot generated (false positive). Interestingly, the human reviewers stated that it was difficult to identify whether the abstract was written by a human author or a chatbot [1,2].

Another use for chatGPT can be in medical education. A study assessed ChatGPT's ability to handle complex medical and clinical information by testing its performance on the US Medical Licensing Examination (USMLE) Step 1, Step 2 CK, and Step 3, as open-ended and multiple-choice questions (MCQ). In the first, ChatGPT scores range from 43% to 68%, and in the MCQ range from 40% to 65%. The accuracy was lowest on Step 1 of the USMLE, regarded as the most difficult exam. This indicates that the AI's performance is tied to human perception and understanding of the subject matter. High internal concordance was also noticed, especially in correctly answering questions, with a concordance rate of up to 99% [3]. The integration of ChatGPT in academic writing has sparked a polarizing debate among scholars. While some see it as a valuable tool for streamlining the writing process, others view it as a threat to the integrity of authorship [4].

While ChatGPT can write credible scientific essays, the data it generates is a mix of true and completely fabricated ones. This raises concerns about the integrity and accuracy of using large language models in academic writing, such as ChatGPT. We propose that policy and practice for evaluating scientific manuscripts for journals and medical conferences be modified to maintain rigorous scientific standards. We also advocate for including AI output detectors in the editorial process and clear disclosure if these technologies are used. The use of large language models in scientific writing is still debatable regarding ethics and acceptability, together with the potential of creating false experts in the medical field with the potential of causing harm due to a lack of real experience and the generation of expert opinions through AI-ChatGPT.
